# Predicting Renal Denervation Response in Resistant High Blood Pressure by Arterial Stiffness Assessment: A Systematic Review

**DOI:** 10.3390/jcm11164837

**Published:** 2022-08-18

**Authors:** Alexandru Burlacu, Crischentian Brinza, Mariana Floria, Anca Elena Stefan, Andreea Covic, Adrian Covic

**Affiliations:** 1 Faculty of Medicine, University of Medicine and Pharmacy “Grigore T Popa”, 700115 Iasi, Romania; 2 Department of Interventional Cardiology, Institute of Cardiovascular Diseases “Prof. Dr. George I.M. Georgescu”, 700503 Iasi, Romania; 3 Department of Internal Medicine and Cardiology—“St. Spiridon Hospital”, 700111 Iasi, Romania; 4 Dialysis and Renal Transplant Center, Nephrology Clinic, “C.I. Parhon” University Hospital, 700503 Iasi, Romania

**Keywords:** resistant arterial hypertension, renal denervation, responders, non-responders, prediction

## Abstract

Background: Accurately selecting hypertensive candidates for renal denervation (RDN) therapy is required, as one-third of patients who undergo RDN are non-responders. We aimed to systematically review the literature on RDN response prediction using arterial stiffness assessment, optimizing the selection of patients referred for interventional blood pressure lowering procedures. Methods: A literature search was performed in MEDLINE, Embase, Scopus, and Cochrane databases to retrieve potential eligible studies from the inception to 30 June 2022. Results: Ten studies were finally included in this systematic review. Studies consistently documented that invasive pulse wave velocity (PWV) was correlated with RDN’s significant success. Nevertheless, non-invasive ambulatory arterial stiffness index and PWV derived from ambulatory blood pressure monitoring were independent predictors of blood pressure response (*p* = 0.04 and *p* < 0.0001). In some studies, magnetic resonance imaging parameters of arterial stiffness (ascending aortic distensibility, total arterial compliance) were correlated with blood pressure reduction (AUC = 0.828, *p* = 0.006). Conclusions: Assessing arterial stiffness prior to RDN predicted procedural success, since stiffness parameters were strongly correlated with a significant blood pressure response. Our endeavor should be tackled as a step forward in selecting appropriate hypertensive patients scheduled for RDN therapy. Non-invasive measurements could be an alternative to invasive parameters for response prediction.

## 1. Introduction

Arterial hypertension (AHT) exerts a significant burden on public health, as it constitutes one of the most critical risk factors for cardiovascular disease (CVD) and death globally [1]. Almost one-third of the population from low- and middle-income countries is diagnosed with AHT, with a slightly lower proportion in high-income countries (28.5%). Moreover, the control rate of hypertension is still low (47.3 ± 1.17%), despite current therapeutic strategies [1,2].

According to the latest European and American guidelines, resistant AHT is defined as persistent increased blood pressure despite optimal drug therapy with at least three antihypertensives (including a diuretic) [3,4]. Although the prevalence of resistant hypertension is lower (15% of subjects treated for AHT), the risk of adverse cardiovascular events is 50% higher than in patients with controlled AHT [5]. A 2018 meta-analysis with a large population sample (*n* = 3,207,911) reported a 10.3% prevalence of true-resistant hypertension, which was even higher in the presence of chronic kidney disease (CKD, 22.9%) or older age (12.3%) [6].

Clinical trials consistently documented an increased risk of cardiovascular events in patients with resistant AHT. During a follow-up of 3.8 years, these patients expressed an increased risk of death, myocardial infarction, heart failure, stroke, and CKD (HR 1.47, 95% CI, 1.33–1.62, *p* < 0.001). Compared to patients with controlled AHT, those with resistant AHT exerted a 2.5-fold higher risk of adverse cardiovascular events [7]. Moreover, cardiovascular mortality was increased by 47% compared to participants with non-resistant AHT. Furthermore, all-cause mortality increased by 33% in patients with uncontrolled AHT. Therefore, appropriate treatment of resistant cases is mandatory to improve long-term outcomes [8].

Besides drug therapy, interventional procedures are available for lowering blood pressure. Previous European guidelines on hypertension management recommended renal denervation intervention (RDN) in patients refractory to drug therapy (class IIb recommendation, level of evidence C) [9]. However, the 2018 guidelines did not advocate for the routine use of device-based therapies [3]. Data regarding RDN efficacy are discrepant in the literature. One of the key studies in the field is the SIMPLICITY HTN-3 trial (randomized, single-blind, sham-controlled trial). The authors did not observe any differences in blood pressure reduction between the groups at six months of follow-up (*p* = 0.98), with similar safety profiles [10]. Using first-generation RDN systems could represent a potential explanation for SIMPLICITY HTN-3 trial neutral results. New-generation RDN systems appear to be consistently efficient and safe for blood pressure reduction [11,12]. A recent meta-analysis of nine randomized sham-controlled trials reported contrasting yet noteworthy results [13]. RDN reduced not only 24 h ambulatory systolic blood pressure (SBP, *p* < 0.001) but also daytime SBP (*p* < 0.001), nighttime SBP (*p* = 0.006), and office SBP (*p* < 0.001) [13].

The matter might not reside exclusively in trying to confirm the superiority of RDN versus standard drug therapy but in identifying the appropriate subgroup of patients that would benefit from the interventional procedure. This argument is also the main reason for our current paper. The (so-called) markers of RDN response could prove efficient in selecting patients. Higher baseline blood pressure, larger renal artery diameter, and higher baseline office heart rate were associated with a significant blood pressure reduction following RDN [14,15]. Additionally, few trials advocated arterial stiffness as a possible predictor of RDN [16].

The concept of arterial stiffness refers to the arteries’ elasticity, distensibility, and compliance proprieties [17,18]. The balance between arterial wall elastin and collagen constitutes a major determinant of arterial stiffness. Arterial stiffness increases once the balance is disrupted due to elastin degeneration or collagen accumulation [17]. The contribution of arterial rigidity might prevail in AHT patients with increased arterial stiffness. Thus, RDN could fail to reduce blood pressure in this subset of patients [16].

Pulse wave velocity (PWV) is a strong marker of arterial stiffness (though the terms are not synonymous), being related to arterial wall distensibility [17]. Moreover, carotid-femoral PWV is a guidelines’ recommendation class I, level of evidence A for arterial stiffness evaluation [19]. Thereby, it should be explored if arterial stiffness could help to identify patients who are likely or unlikely to respond to RDN therapy.

Therefore, we aimed to systematically review the literature on RDN response prediction using arterial stiffness to optimize the selection of patients referred for interventional blood-pressure-lowering procedures.

## 2. Materials and Methods

For standardized reporting, the present systematic review was conducted according to the updated Preferred Reporting Items for Systematic Review and Meta-Analyses (PRISMA) guidelines [20]. The study protocol was registered in the PROSPERO database (CRD42022348207).

### 2.1. Data Sources and Search Strategy

A comprehensive literature search was performed in MEDLINE (PubMed), Embase, Scopus, and Cochrane databases to retrieve potential eligible studies from the inception to 30 June 2022. No language restrictions or filters were applied in the search process. We also screened references from cited articles, the Google Scholar search engine, and the ClinicalTrials.gov database of clinical trials, as endorsed by the PRISMA checklist. Combinations between the following keywords and MeSH terms (for MEDLINE database) or Emtree terms (for Embase database) were used to build a search strategy: “arterial hypertension”, “resistant hypertension”, “uncontrolled hypertension”, “high blood pressure”, “renal sympathetic denervation”, “renal denervation”, “response”, “responders”, “prediction”, “arterial stiffness”, and “pulse wave velocity”. The search strategy for each database and the retrieved studies were reported in Table S1.

### 2.2. Eligibility Criteria and Outcomes

Two independent investigators carried out the eligibility assessment of retrieved studies based on pre-established inclusion and exclusion criteria. Studies were considered for inclusion in the present systematic review if they fulfilled the following inclusion criteria: (1) observational studies or randomized clinical trials; (2) participants aged ≥18 years with AHT who underwent RDN were enrolled; (3) arterial stiffness was appraised invasively or non-invasively prior to RDN procedure; and (4) original data were reported concerning the association between arterial stiffness and response to RDN during follow-up (decreased 24 h blood pressure, SBP and diastolic blood pressure—DBP). In addition, critical exclusion criteria were set to guide the eligibility assessment: unpublished data, studies with overlapping populations, editorials, meta-analysis, case reports, and missing data or inability to extract data.

### 2.3. Data Collection and Synthesis

The following data were extracted from eligible studies that met the inclusion criteria: first author, publication year, population sample size, age of enrolled participants, clinical setting, methods of arterial stiffness measurement, reported outcomes, and follow-up period. Moreover, essential inclusion and exclusion criteria of individual studies that could affect outcome interpretation were extracted and critically analyzed. Data were presented as median or mean values, the area under the curve (AUC), odds ratio (OR), and *p*-values whenever available.

### 2.4. Quality Assessment

The quality of included studies was assessed according to their design. The risk of bias in randomized clinical trials was appraised using the revised Cochrane risk-of-bias tool for randomized trials (RoB 2) [21]. In the case of observational non-randomized studies, the Newcastle–Ottawa scale (NOS) was applied to judge the overall quality of the studies. It consists of several essential signaling questions, addressing three domains (population sample selection, comparability of groups, and outcomes evaluated) [22].

## 3. Results

**Figure 1:**
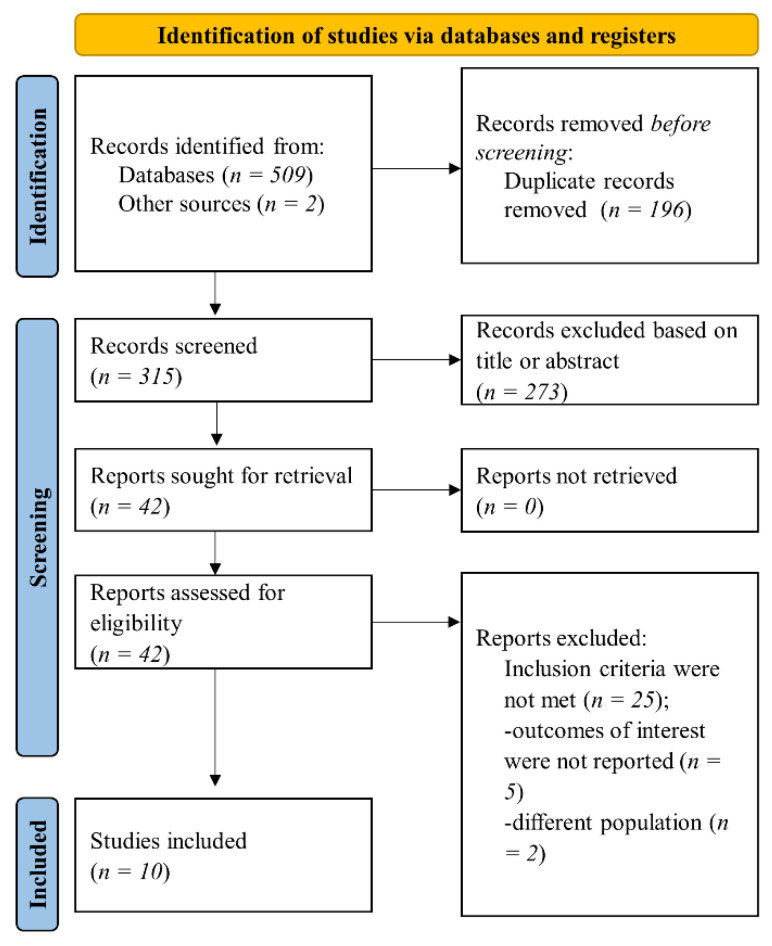
Flow diagram of selected studies in the present analysis.

Our search in pre-specified databases and sources retrieved 511 references. Afterward, duplicate records were excluded. The remaining 315 articles were initially screened for eligibility based on title or abstract, and 273 records were excluded. In the next step, 42 records were assessed in full text for inclusion and exclusion criteria. Finally, 10 studies that fulfilled the inclusion criteria were included in the present systematic review (Figure 1).

General data from analyzed studies, including population sample size, age of enrolled patients, clinical setting, outcomes, and follow-up duration, are provided in Table 1. Moreover, arterial stiffness measurement methods and RDN response definition used in individual studies (when available) are reported in Table 1. The association between the investigated arterial stiffness parameters (invasive or non-invasive) and the outcomes evaluated in clinical studies is displayed in Table 2.

The majority of included studies had an observational design [16,23,24,25,26,27,29,30], while only two studies were performed as secondary analyses from randomized clinical trials [28,31].

Definitions of variables and methods used were different across studies. Of the included studies, five measured arterial stiffness exclusively by non-invasive methods: PWV, central pulse pressure, ambulatory arterial stiffness index (AASI), and magnetic resonance-derived parameters [23,28,29,30,31]. Definition of blood pressure response to RDN also varied in clinical studies; some used a 5 mmHg cut-off [16,24,25,26], while the others used a 10 mmHg cut-off or 5% decrease to delineate between responders and non-responders [29,30].

Data on non-invasive PWV measurement were available from three studies [16,28,31]. Moreover, five reported data on invasive PWV assessment [16,24,25,26]. Non-invasive PWV was measured using magnetic resonance imaging only in one study [16], while the other two investigated classic non-invasive PWV [28,31].

In one of the two studies investigating classic non-invasive PWV, the authors reported that estimated PWV from ambulatory blood pressure monitoring was independently associated with blood pressure response at multivariate analysis (OR 0.031, 95% CI, 0.006–0.167, *p* < 0.0001) [31]. Although estimated PWV was independently associated with RDN response, the proportion of responders stratified according to PWV values was not reported. Moreover, estimated PWV, augmentation pressure, backward wave amplitude, and forward wave amplitude had modest predictive power for RDN response, with AUC ranging from 0.62 (95% CI, 0.53–0.71) to 0.74 (95% CI, 0.64–0.82) [31]. However, classic non-invasive PWV measurement was not associated with RDN response at follow-up in the second study. Nevertheless, the small sample size (*n* = 53) limits the application of the results in all AHT patients [28].

The most recent trial investigated response prediction to RDN using invasive PWV compared to non-invasive markers (ascending aortic distensibility, PWV, and total arterial compliance derived from magnetic resonance imaging) [16]. Invasive PWV and ascending aortic distensibility measured non-invasively were documented as independent predictors of blood pressure response to RDN (*p* = 0.019 and *p* = 0.006). However, PWV measured by magnetic resonance imaging was not correlated with blood pressure drop at univariate analysis (*p* = 0.07) [16].

In terms of cut-off values, invasive PWV < 14.4 m/s was linked to a better blood pressure response than invasive PWV above the established value (*p* < 0.01) [16]. Notably, the predictive power of ascending aortic distensibility (measured non-invasively) was somewhat better than for invasive PWV (AUC 0.714 and 0.695, respectively). Furthermore, integrating arterial stiffness variables in a bivariate model (logarithmic ascending aortic distensibility and baseline 24 h SBP) or a multivariable model significantly improved the predictive accuracy of blood pressure response to RDN (bivariate model: AUC 0.740; multivariate model: AUC 0.791) [16].

Another study compared non-invasive arterial stiffness measurement using magnetic resonance imaging with invasive PWV [26]. Invasive PWV and ascending aortic distensibility had good predictive power (AUC 0.849 and AUC 0.828). Noteworthy, in multivariate analysis, only ascending aortic distensibility assessed by magnetic resonance imaging was linked to RDN response (OR 6.8, 95% CI, 1.4–34.2, *p* = 0.019). Other parameters failed to prove significant in multivariate analysis [26]. The authors from the other two studies documented that low PWV measured invasively was associated with blood pressure response to RDN [24,25]. Moreover, a 13.7 m/s cut-off for invasive PWV had 71% sensibility, 83% specificity, and 85.7% positive predictive value for RDN response [24].

Another study investigated the association between invasive PWV or non-invasive pulse pressure with blood pressure drop following RDN [26]. The authors reported a statistically significant association with blood pressure reduction only in the case of invasive PWV at multivariate analysis (OR 0.834, 95% CI, 0.724–0.961, *p* = 0.012). Pulse pressure measured non-invasively was not associated with blood pressure response (*p* = 0.16). However, response to RDN was defined as a drop ≥ 20 mmHg in ambulatory daytime average blood pressure, which differed from other analyzed studies. Therefore, the study’s methodology might affect the results and should be considered in case of extrapolation to other patients [26].

Unconvincing results regarding pulse pressure and blood pressure response were obtained in another study [23]. Low central pulse pressure measured non-invasively was associated with a reduction in office blood pressure values compared to high central pulse pressure (*p* = 0.038 for SBP and *p* = 0.014 for DBP). Although 24 h blood pressure drop was slightly bigger in subgroup with low central pulse pressure, it did not reach statistical significance (*p* = 0.07 for 24 h SBP and *p* = 0.112 for 24 h DBP) [23].

AASI derived from 24 h ambulatory blood pressure monitoring was associated with RDN response, as was documented in one study [29]. AASI lower than 0.51 was linked to blood pressure drop even after adjustment for multiple variables (OR 3.46, 95% CI, 1.0–13.3, *p* = 0.04). When the AASI cut-off was set at 0.64, it had a 100% sensitivity, 29% specificity, 32% positive predictive value, and 13% negative predictive value for RDN response [29].

**Figure 2:**
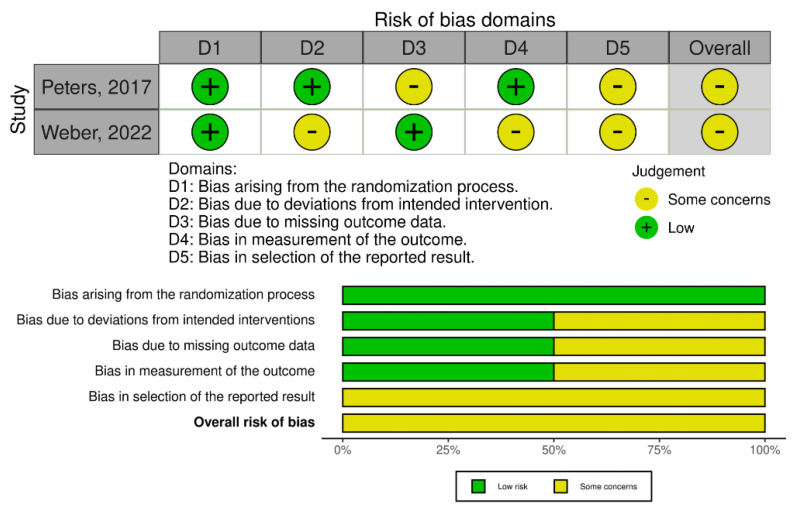
The overall risk of bias assessment using the revised Cochrane risk-of-bias tool [28,31].

The overall quality of analyzed observational studies was modest to good, as appraised using the NOS scale (Table S2). There were some concerns regarding the risk of bias in the case of randomized trials (post hoc analyses) evaluated by the RoB 2 tool (Figure 2).

## 4. Discussion

To the best of our knowledge, this systematic review is the first to investigate reported data in the literature on the validity of arterial stiffness for blood pressure response prediction following RDN.

Our endeavor should be perceived as a step forward in selecting those patients with resistant hypertension who most likely will respond to RDN therapy. Moreover, it certainly is a background for future studies and clinical models to increase the discriminatory capacity between responders and non-responders to RDN. Arterial stiffness could improve cardiovascular risk stratification in AHT patients who are candidates for RDN, as it is associated with adverse cardiovascular events and all-cause mortality [32]. In addition, arterial stiffness could be measured early and late after RDN to identify the potential (arterial) ‘destiffening’ with subsequent impact on overall cardiovascular risk [33].

The guidelines describe and endorse several methods to evaluate arterial stiffness [19]. Non-invasive assessment strategies usually include pulsed-wave velocity evaluation and parameters derived from magnetic resonance imaging.

Non-invasive PWV is a ‘marker of arterial stiffness’ referred to as the ‘gold standard’ of arterial stiffness measurement [18]. Notably, carotid-femoral non-invasively PWV received a guidelines’ recommendation class I, level of evidence A for arterial stiffness evaluation. Moreover, PWV could be measured in other arterial places, including ankle-brachial index or cardiac-ankle stiffness index (class I, level of evidence B). Devices and approaches to evaluate PWV are also provided by the guidelines: e.g., devices using a tonometer, oscillometric devices, or those using an ultrasound probe [19].

Magnetic resonance measurement could also acquire non-invasive data regarding blood flow velocity as well as arterial distensibility and compliance, with good technical reproducibility [19]. Moreover, a good correlation between magnetic-resonance-derived PWV and invasive PWV was reported [34]. Data suggested similar values of non-invasive parameters compared to invasive methods [34].

Invasive aortic PWV assessment constitutes an accurate and reproducible tool to evaluate arterial stiffness. Nevertheless, this ‘old-fashioned’ intravascular PWV assessment might not provide additional (and clinically significant) information compared to non-invasive assessment. In addition, invasive PWV evaluation has limited applicability in the general population due to its invasiveness and potential complications [19].

Though randomized clinical trials documented a benefit in blood pressure reduction after RDN, almost one-third of patients who underwent RDN were non-responders [35,36]. In other words, up to a third of patients with hypertension who had renal denervation did not respond to the procedure. In the era of targeted therapies, non-invasive markers of RDN response are particularly interesting. It has been suggested that baseline ambulatory daytime DBP, number of antihypertensive drugs administered, or orthostatic hypertension could predict the response to RDN [36].

However, none of the investigated predictive parameters could accurately identify all patients who would benefit from RDN [36]. New markers were developed and validated to increase the predictive accuracy, such as higher baseline blood pressure, larger renal artery diameter, or higher baseline office heart rate [14,15]. In this regard, arterial stiffness measurement prior to RDN attracted interest in the last decade. Several clinical studies reported that arterial stiffness measurements could improve the selection of patients responding to RDN [23,24]. Patients with lower arterial stiffness parameters were prone to respond, as 10 patients out of 13 were responders, while in the subgroup with increased arterial stiffness, only 3 patients out of 13 responded to RDN. Consequently, measuring arterial stiffness could identify 77% of patients who would probably respond to RDN [25]. In this case, arterial stiffness had a modest to good predictive power, with AUC up to 0.849 [26].

On another note, arterial stiffness could also distinguish that one-third of non-responder patients were unlikely to exhibit a blood-pressure reduction. Most patients with AHT and increased arterial stiffness were non-responders (84%), as highlighted in one study [29]. Consequently, a significant proportion of patients who are unlikely to respond could be spared from worthless complications and exposure to an invasive and radiating procedure. RDN is cost-effective when performed only in a certain subgroup of high-risk patients [37]. Assessment of arterial stiffness prior to RDN could identify 84% of patients who would be unlikely to respond, avoiding a futile invasive procedure [29]. Thus, optimizing the selection of patients for RDN (also by arterial stiffness measurement) improves the cost-efficiency ratio.

As arterial stiffness could be evaluated non-invasively, it represents a feasible marker that could be implemented in clinical practice. Ascending aortic distensibility measured non-invasively by magnetic resonance imaging performed slightly better in predicting RDN response than invasively PWV (AUC 0.714 vs. AUC 0.695). Accordingly, ascending aortic distensibility could accurately distinguish between responders and non-responders in 71% of cases, which was improved by integrating non-invasive markers in different prediction models [16]. In addition, augmentation pressure had similar discrimination power, allowing an accurate response prediction in 74% of patients (*p* < 0.0001) [31].

In combination with other clinical and paraclinical parameters, arterial stiffness could be included in a multivariate prediction model further to refine the selection of patients [16]. Proposed bivariate/multivariate models significantly improved the ability to discriminate between responders and non-responders (AUC 0.740 and 0.791, respectively). Subsequently, a multivariate model could predict blood pressure response in almost 80% of cases. It seems reasonable to integrate additional response markers rather than perform or preclude RDN based on a single marker approach [16].

A potential explanation for the association between arterial stiffness and RDN response relies on AHT and RDN pathophysiology [16]. RDN influences the neurohormonal component of AHT, including sympathetic nervous system activation [16,38]. However, arterial stiffness increases with age; thus, a biomechanical component of AHT could prevail over sympathetic activity in this subgroup of patients. Moreover, aortic stiffness is usually caused by the destruction of elastin in the aortic wall and substitution with fibrosis. At that stage, it could probably be too late to intervene, as the rigidity of fibrotic arteries perpetuates AHT rather than sympathetic-induced vascular smooth muscle cell contraction [16,17,39,40]. In consequence, the effect of RDN on the biomechanical part of AHT and, subsequently, on blood pressure reduction could be limited in patients with increased arterial stiffness [16].

Nevertheless, non-invasive measurement of arterial stiffness is susceptible to different physiological and methodological confounders, which should be considered when implementing arterial stiffness in clinical practice or research [19]. Mean arterial pressure constitutes a vital confounder, as stated in the American Heart Association (AHA) scientific statement on improving and standardizing vascular research on arterial stiffness [19]. In addition, the increased heart rate could be linked to higher arterial stiffness, especially in patients with increased mean arterial pressure. Moreover, the lack of PWV measurement standardization across different healthcare centers could limit the discrimination power between responders and non-responders to RDN. Therefore, arterial stiffness should be assessed in a standardized context and environment, in line with AHA recommendations [19]. Adopting a protocol for arterial stiffness measurement, especially for the purpose of research, could enhance the reproducibility and robustness of obtained results.

Notably, sodium–glucose co-transporter-2 (SGLT2) inhibitors could also alleviate arterial stiffness parameters and the blood pressure reduction effect [41,42]. Therefore, SGLT2 inhibitors could affect the arterial stiffness measurement prior to RDN. The possibility of influencing outcomes following RDN by SGLT2 inhibitors therapy should be investigated in clinical trials.

Another limitation derives from the RDN techniques performed. RDN could be achieved by applying three distinct ablation types: radiofrequency, ultrasound, and alcohol-mediated ablation. In our systematic review, radiofrequency ablation was used in most clinical studies [23,24,26,28,29,30,31], while only three used radiofrequency and ultrasound techniques [16,25,27]. Moreover, none of the studies explored alcohol-mediated RDN. Thus, caution is required when extrapolating the results across all RDN techniques.

## 5. Conclusions

Arterial stiffness constitutes a significant part of the solution for selecting appropriate hypertensive patients for renal denervation intervention. Accurately selecting candidates for RDN is required, as almost one-third of patients who undergo RDN are non-responders. The solution provided by assessing arterial stiffness is attractive, as it could be measured non-invasively—a standing alternative to invasive parameters for response prediction. As reported in clinical studies, arterial stiffness parameters were strongly correlated with a greater blood pressure response to RDN. The ability of arterial stiffness to discriminate between responders and non-responders was good in all analyzed studies. Arterial stiffness could be integrated with other (clinical and paraclinical) parameters as part of a multivariate prediction model to refine further the selection of patients who would benefit from RDN. Therefore, this systematic review should be tackled as a step forward in selecting appropriate AHT patients scheduled for RDN therapy. More standardized and robust data is required before introducing arterial stiffness as a major predictor of RDN response, due to heterogeneity in the methodology, RDN techniques, and center experience across studies.

## Figures and Tables

**Table 1:** General characteristics of studies included in the present systematic review.

First Author, Year	Design	Patients, No	Age, Median/Mean ± SD	Setting	Methods	Outcomes	Follow-Up
Ott et al., 2015 [23]	Observational, prospective, single-center	63	56.5 ± 11 (low cPP)	Patients with TRH (office BP ≥ 140/90 mmHg and 24 h ABP ≥ 130/80 mmHg despite treatment with at least 3 AHT drugs, including a diuretic) and eGFR ≥ 15 mL/min/1.73 m^2^.	Baseline cPP was measured using SphygmoCor. Patients were stratified according to median cPP: low cPP (below 55 mmHg) and high cPP (above 55 mmHg). RDN—radiofrequency technique.	(a) Office and 24 h systolic and diastolic BP reduction after RDN. (b) Renal function.	6 months
			66.1 ± 8.0 (high cPP)				
Okon et al., 2016 [24]	Observational, single-center	58	60.41 ± 10.3 (responders)	Patients with resistant hypertension (24 h ABP: mean daytime systolic BP ≥ 135 mmHg or diastolic BP ≥ 90 mmHg, despite treatment with at least 3 AHT drugs, including a diuretic. Patients with eGFR < 45 mL/min/1.73 m^2^ were excluded.	PWV was measured invasively. RDN response was defined as reduction with ≥5 mmHg in systolic daytime BP (24 h ABPM). RDN—radiofrequency technique.	Daytime, night-time, and 24 h BP reduction after RDN.	6 months
			63.1 ± 9.0 (non-responders)				
Fengler et al., 2017 [25]	Observational, prospective, single-center	109	60.4 ± 9.0 (combined hypertension)	Patients with resistant hypertension, defined as mean daytime systolic BP > 135 mmHg or diastolic BP > 90 mmHg in ABPM despite treatment with at least 3 AHT drugs, including 1 diuretic unless intolerant.	PWV was measured invasively immediately before renal denervation. Response to RDN was defined as a drop ≥ 5 mmHg in ABPM daytime systolic BP after 3 months. RDN—radiofrequency and ultrasound techniques.	(a) BP reduction after RDN at 3 months. (b) BP response in relation to PWV tertiles.	3 months
			66.5 ± 9.8 (isolated systolic hypertension)				
Fengler et al., 2018 [26]	Observational, single-center, study sub-analysis	32	64.5 ± 9.9	Patients treated for resistant hypertension, defined as mean daytime systolic ≥135 mmHg or diastolic BP ≥ 90 mmHg in ABPM, despite intake of at least 3 AHT drugs, including a diuretic. Patients with eGFR < 45 mL/min/1.73 m^2^ were excluded.	Arterial stiffness measured using MRI (ascending aortic distensibility, total arterial compliance, systemic vascular resistance) versus invasive PWV. Response to RDN was defined as a drop ≥5 mmHg in ABPM daytime systolic BP after 3 months. RDN—radiofrequency technique.	(a) BP reduction after RDN using ABPM. (b) Invasive and non-invasive parameters of arterial stiffness as predictors for the response after RDN.	3 months
Fengler et al., 2022 [16]	Observational, prospective, single-center	79	62.6 ± 8.8	Patients with resistant hypertension defined as systolic daytime BP > 135 mmHg, despite treatment with 3 or more different classes of AHT drugs, including one diuretic, unless intolerant to diuretics.	Arterial stiffness was measured invasively (PWV) or non-invasively (CMR-derived ascending aortic distensibility, PWV, and total arterial compliance). Response to RDN was defined as a drop ≥ 5 mmHg in ABPM daytime systolic BP after 3 months. RDN—ultrasound and radiofrequency (in validation cohort) techniques.	(a) Change in systolic daytime BP on ABPM at 3 months in different arterial stiffness subgroups. (b) RDN response predicting power of non-invasive arterial stiffness parameters compared to invasive PWV measurement.	3 months
Fengler et al., 2018 [27]	Observational, retrospective, single-center	190	62.2 ± 9.9	Patients with TRH defined as office systolic BP > 160 mmHg and 24 h BP > 135/90 mmHg, despite treatment with 3 or more classes of AHT drugs, including one diuretic, unless intolerant to diuretics.	PWV measured invasively and non-invasive pulse pressure. Response to RDN was defined as a drop ≥ 5 mmHg in ABPM daytime average BP after 3 months. The profound response was defined as a drop ≥ 20 mmHg in ABPM daytime average BP. RDN—radiofrequency and ultrasound techniques.	Change in BP on ABPM, including a profound response, in relation to arterial stiffness.	3 months
Peters et al., 2017 [28]	Substudy of a randomized, sham-controlled, double-blind trial	53	59 ± 9 (sham)	Patients with therapy-resistant hypertension, with daytime ABPM systolic >145 mmHg and 1 month of stable treatment with at least 3 AHT drugs, including a diuretic. Patients with eGFR < 30 mL/min/1.73 m^2^ were excluded.	Carotid-femoral PWV was measured non-invasively at baseline and after 6 months (SphygmoCor). RDN—radiofrequency technique.	Changes in 24 h AMBP and PWV after RDN.	6 months
			54 ± 8 (RDN)				
Sata et al., 2018 [29]	Observational, retrospective	111	63.2 ± 10.3	Patients with resistant hypertension are defined as having office BP > 140/90 mmHg, despite prescribed treatment with three or more AHT drugs.	The ambulatory arterial stiffness index was derived from 24 h ABPM monitoring. Response to RDN was defined as a reduction of 5% in systolic BP on ABPM. RDN—radiofrequency technique.	(a) Reduction in systolic BP on ABPM after 6 months from RDN. (b) The predictive value of RDN response attributed to ambulatory arterial stiffness index.	12 months
Stoiber et al., 2018 [30]	Observational, prospective, multicenter	58	64.4 ± 9.6	Resistant hypertension was defined as office systolic BP ≥ 140 mmHg or mean ambulatory 24 h systolic BP > 135 mmHg despite using≥ 3 AHT drugs, including a diuretic.	Aortic distensibility was derived from MRI. Response to RDN was defined as reduction with at least 10 mmHg in systolic BP. RDN—radiofrequency technique.	(a) Office systolic and diastolic BP at 6 months after RDN in relation to aortic distensibility. (b) Aortic distensibility response to RDN.	6 months
Weber et al., 2022 [31]	A post hoc analysis of a randomized, sham-controlled clinical trial	222	53.0 ± 11.0 (RDN)	Patients with average systolic BP ≥ 140 mmHg and <170 mmHg on 24 h ABPM, office systolic BP ≥ 150 mmHg and <180 mmHg, and office diastolic BP ≥ 90 mmHg.	Augmentation index, augmentation pressure, backward and forward wave amplitude, estimated aortic PWV, measured non-invasively. RDN—radiofrequency technique.	Predictive value of RDN response in relation to non-invasive arterial stiffness parameters.	3 months
			51.6 ± 11.0 (sham)				

ABPM = ambulatory blood pressure monitoring; AHT = antihypertensive; BP = blood pressure; cPP = central pulse pressure; eGFR = estimated glomerular filtration rate; MRI = magnetic resonance imaging; PWV = pulse wave velocity; RDN = renal denervation; TRH = treatment resistant hypertension.

**Table 2:** Results reported in clinical studies included in the present systematic review.

Study, Year	Parameters	Outcomes	Results		
Ott, 2015 [23]			Pre-RDN	Post-RDN	
	Low cPP	Office SBP, mmHg	160 ± 16	137 ± 16	*p* < 0.001
		Office DBP, mmHg	95 ± 13	82 ± 11	*p* < 0.001
		24 h SBP, mmHg	155 ± 15	144 ± 15	*p* < 0.001
		24 h DBP, mmHg	93 ± 12	86 ± 10	*p* < 0.001
		eGFR, mL/min/1.73 m^2^	76.4 ± 21	76.0 ± 22	*p* = 0.846
	High cPP	Office SBP, mmHg	166 ± 20	154 ± 26	*p* = 0.003
		Office DBP, mmHg	85 ± 16	80 ± 14	*p* = 0.049
		24 h SBP, mmHg	157 ± 16	154 ± 23	*p* = 0.326
		24 h DBP, mmHg	84 ± 11	81 ± 12	*p* = 0.059
		eGFR, mL/min/1.73 m^2^	72.1 ± 28	70.1 ± 30	*p* = 0.243
	cPP	Office SBP reduction, mmHg	−22 ± 19 in low cPP vs.−12 ± 20 in high cPP		*p* = 0.038
		Office DBP reduction, mmHg	−13 ± 11 in low cPP vs.−5 ± 13 in high cPP		*p* = 0.014
		24 h SBP reduction, mmHg	−11 ± 13 in low cPP vs.−3 ± 18 in high cPP		*p* = 0.07
		24 h DBP reduction, mmHg	−8 ± 10 in low cPP vs.−4 ± 10 in high cPP		*p* = 0.112
Okon, 2016 [24]	iPWV	RDN response	OR 1.15 (95% CI, 1.014–1.327)		*p* = 0.03
			AUC 0.79 (95% CI, 0.658–0.882)		*p* < 0.0001
			13.7 m/s cut-off: sensitivity 71%, specificity 83%, positive predictive value 85.7%		
Fengler, 2017 [25]	iPWV	Daytime BP response	Patients with iPWV < 14.4 m/s had a better BP response vs. those with iPWV > 14.4 m/s (11.7 ± 12.7 mmHg vs. 7.2 ± 10.4 mmHg)		*p* = 0.047
			Patients with isolated systolic hypertension in the lowest iPWV tertile had the best BP response vs. those in the middle iPWV tertile		*p* = 0.012
			Patients with isolated systolic hypertension in the lowest iPWV tertile had the best BP response vs. those in high iPWV tertile		*p* = 0.013
		Responder rate	77% in low iPWV tertile, 50% in middle iPWV tertile and 23% in high iPWV tertile		*p* = 0.001
		BP response	Per 1 m/s of iPWV: OR 0.91, 95% CI, 0.83–0.99)		*p* = 0.037
Fengler, 2018 [30]	iPWV	BP response	Patients with iPWV < 13.6 m/s had better BP response than those with iPWV > 13.6 m/s (−13.0 ± 8.7 mmHg vs. −4.1 ± 5.5 mmHg)		*p* = 0.002
			AUC 0.849, 95% CI, 0.713–0.985		*p* = 0.004
	AAD	BP response	Patients with AAD above the median (2.0 × 10^−3^ mmHg^−1^) had a better BP response than those with AAD below the median (−11.9 ± 6.9 mmHg vs. −5.6 ± 8.8 mmHg)		*p* = 0.034
			AUC 0.828, 95% CI, 0.677–0.979		*p* = 0.006
			Multivariate analysis: OR 6.8, 95% CI, 1.4–34.2—AAD the only predictor for BP response		*p* = 0.019
	cTAC, TAC	BP response	Patients with cTAC or TAC above the median had a better BP response than those with parameters below the median (−11.6 ± 6.8 mmHg vs. −5.5 ± 9.1 mmHg)		*p* = 0.041
	cTAC	BP response	AUC 0.776, 95% CI, 0.563–0.989		*p* = 0.021
	TAC	BP response	AUC 0.753, 95% CI, 0.576–0.929		*p* = 0.035
Fengler, 2022 [16]	iPWV	Daytime BP reduction	β 0.242, 95% CI, 0.054–0.430		*p* = 0.012
		24 h BP reduction	β = 0.232, 95% CI, 0.046–0.419, AUC 0.695		*p* = 0.015
	AAD	24 h BP reduction	β = −0.243, 95% CI, −0.428 to −0.058, AUC 0.714		*p* = 0.011
	AAD (logarithmic)	24 h BP reduction	Β = −0.306, 95% CI, −0.484 to −0.128		*p* = 0.001
	TAC	24 h BP reduction	β = −0.058		*p* = 0.61
	PWV (MRI)	24 h BP reduction	β = 0.207		*p* = 0.07
	Carotid-femoral PWV	24 h BP reduction	β = 0.109		*p* = 0.34
Fengler, 2018 [27]	iPWV	BP reduction	Lower iPWV was associated with a higher rate of profound BP response (per m/s: OR 0.834, 95% CI, 0.724–0.961)		*p* = 0.012
	Non-invasive pulse pressure	BP reduction	No differences were observed between no or regular BP response as compared to those with profound BP response		*p* = 0.16
Peters, 2017 [28]	PWV	SBP 24 h response	r^2^ = 0.002		*p* = NS
		MAP reduction	r^2^ = 0.001		*p* = NS
Sata, 2018 [29]	AASI	BP response	Responders had lower AASI compared to non-responders (0.47 ± 0.12 vs. 0.54 ± 0.15)		*p* = 0.031
			84% of patients from the highest AASI tertile were non-respondent, compared to 42% in the lowest AASI tertile		
	AASI < 0.51	BP response	OR 2.62, 95% CI, 1.05–6.79 (univariate analysis)		*p* = 0.038
			OR 3.46, 95% CI, 1.0–13.3 (multivariate adjustment)		*p* = 0.04
	AASI < 0.64	BP response	OR 14.0, 95% CI, 2.57–261.37		*p* = 0.001
Stoiber, 2018 [30]	Aortic distensibility	SBP reduction	−24.0 ± 26.5 mmHg (low distensibility group) vs. −18.5 ± 16.1 mmHg (high distensibility group)		*p* = 0.770
		DBP reduction	−8.4 ± 14.7 mmHg (low distensibility group) vs. −6.9 ± 9.6 mmHg (high distensibility group)		*p* = 0.570
Weber, 2022 [31]	Augmentation index	24 h SBP reduction	−8.4 mmHg in the low augmentation index group vs. −0.6 mmHg in the high augmentation index group		*p* < 0.001
			AUC 0.70, 95% CI, 0.61–0.79		*p* < 0.0001
	Augmentation pressure	24 h SBP reduction	−8.5 mmHg in the low augmentation pressure group vs. −0.5 mmHg in the high augmentation pressure group		*p* < 0.001
			AUC 0.74, 95% CI, 0.64–0.82		*p* < 0.0001
	BWA	24 h SBP reduction	−7.9 mmHg in low BWA group vs. −1.1 mmHg in high BWA group		*p* < 0.001
			AUC 0.70, 95% CI, 0.61–0.79		*p* < 0.0001
	FWA	24 h SBP reduction	−7.4 mmHg in low FWA group vs. −1.7 mmHg in high FWA group		*p* = 0.004
			AUC 0.65, 95% CI, 0.55–0.74		*p* = 0.004
	ePWV	24 h SBP reduction	−8.4 mmHg in low ePWV group vs. −0.6 mmHg in high ePWV group		*p* < 0.001
			AUC 0.62, 95% CI, 0.53–0.71		*p* = 0.03

AAD = ascending aortic distensibility; AASI = ambulatory arterial stiffness index; AUC = area under the curve; BP = blood pressure; BWA = backward wave amplitude; cPP = central pulse pressure; cTAC = central pressure total arterial compliance; DBP = diastolic blood pressure; ePWV = estimated aortic pulse wave velocity; FWA = forward wave amplitude; iPWV = invasive pulse wave velocity; MAP = mean arterial blood pressure; MRI = magnetic resonance imaging; NS = nonsignificant; RDN = renal denervation; SBP = systolic blood pressure; TAC = total arterial compliance.

## Supplementary Materials

The following supporting information can be downloaded at: https://www.mdpi.com/article/10.3390/jcm11164837/s1. Table S1: Databases and search strategy; Table S2: Quality assessment.
