# Supplementary material for: Predicting Renal Denervation Response in Resistant High Blood Pressure by Arterial Stiffness Assessment: A Systematic Review
Source: J Clin Med. 2022 Aug 18;11(16):4837. doi: 10.3390/jcm11164837 (PMC9410368; doi:10.3390/jcm11164837)
Supplement: Supplementary file 1 [file jcm-11-04837-s001.zip › Table S1 Databases and search strategy.pdf]

Table S1. Databases and search strategies used in present systematic review.

Database	Coverage	Search run	Records
**MEDLINE**	1946 to present	"(Arterial hypertension OR resistant hypertension OR uncontrolled hypertension OR high blood pressure) AND (renal sympathetic denervation OR renal denervation) AND (arterial stiffness OR pulse wave velocity) AND (response OR responders OR prediction)"	26
		"(Renal sympathetic denervation OR renal denervation) AND (arterial stiffness OR pulse wave velocity) AND (response OR responders OR prediction)"	27
		"(Renal sympathetic denervation OR renal denervation) AND (arterial stiffness OR pulse wave velocity)"	65
		"Renal sympathetic denervation OR renal denervation) AND (arterial stiffness)"	55
			Total records = 173
**Embase**	1966 to present	"(Arterial hypertension OR resistant hypertension OR uncontrolled hypertension OR high blood pressure) AND (renal sympathetic denervation OR renal denervation) AND (arterial stiffness OR pulse wave velocity) AND (response OR responders OR prediction)"	51
		"(Renal sympathetic denervation OR renal denervation) AND (arterial stiffness OR pulse wave velocity) AND (response OR responders OR prediction)"	0
		"(Renal sympathetic denervation OR renal denervation) AND (arterial stiffness OR pulse wave velocity)"	0
		"Renal sympathetic denervation OR renal denervation) AND (arterial stiffness)"	0
			Total records = 51
**Cochrane library**	1967 to present	"(Arterial hypertension OR resistant hypertension OR uncontrolled hypertension OR high blood pressure) AND (renal sympathetic denervation OR renal denervation) AND (arterial stiffness OR pulse wave velocity) AND (response OR responders OR prediction)"	0
		"(Renal sympathetic denervation OR renal denervation) AND (arterial stiffness OR pulse wave velocity) AND (response OR responders OR prediction)"	0
		"(Renal sympathetic denervation OR renal denervation) AND (arterial stiffness OR pulse wave velocity)"	0
		"Renal sympathetic denervation OR renal denervation) AND (arterial stiffness)"	280
			Total records = 280
**Scopus**	From the inception till present	"(Arterial hypertension OR resistant hypertension OR uncontrolled hypertension OR high blood pressure) AND (renal sympathetic denervation OR renal denervation) AND (arterial stiffness OR pulse wave velocity) AND (response OR responders OR prediction)"	5
		"(Renal sympathetic denervation OR renal denervation) AND (arterial stiffness OR pulse wave velocity) AND (response OR responders OR prediction)"	0

	“(Renal sympathetic denervation OR renal denervation) AND (arterial stiffness OR pulse wave velocity)”	0
	“Renal sympathetic denervation OR renal denervation) AND (arterial stiffness)”	0
		Total records = 5
All databases: 509 records		
