# Supplementary material for: Predicting Renal Denervation Response in Resistant High Blood Pressure by Arterial Stiffness Assessment: A Systematic Review
Source: J Clin Med. 2022 Aug 18;11(16):4837. doi: 10.3390/jcm11164837 (PMC9410368; doi:10.3390/jcm11164837)
Supplement: Supplementary file 1 [file jcm-11-04837-s001.zip › Table S2 Quality assessment.pdf]

Table S2. Quality assessment of included studies using Newcastle-Ottawa scale.

Study	Representativeness of the exposed cohort	Selection of the non-exposed cohort	Ascertainment of exposure	Presence of outcome of interest at the start	Comparability of cohorts	Assessment of outcome	Follow-up long enough for outcomes to occur	Adequacy of follow-up	Total
Ott, 2015	*	*	*	*	*		*		6
Okon, 2016	*	*	*	*	*	*	*	*	8
Fengler, 2017	*	*	*	*	*		*		6
Fengler, 2018	*	*	*	*	*	*	*		7
Fengler, 2022	*	*	*	*	*	*	*	*	8
Fengler, 2018	*	*	*	*	*		*	*	7
Sata, 2018	*	*	*	*	*		*		6
Stoiber, 2018	*	*	*	*	*		*	*	7

Good quality: 3 or 4 stars in selection domain AND 1 or 2 stars in comparability domain AND 2 or 3 stars in outcome/exposure domain. Fair quality: 2 stars in selection domain AND 1 or 2 stars in comparability domain AND 2 or 3 stars in outcome/exposure domain. Poor quality: 0 or 1 star in selection domain OR 0 stars in comparability domain OR 0 or 1 stars in outcome/exposure domain.
